# Hepatic bile acid transport increases in the postprandial state: A functional ^11^C-CSar PET/CT study in healthy humans

**DOI:** 10.1016/j.jhepr.2021.100288

**Published:** 2021-04-15

**Authors:** Nikolaj W. Ørntoft, Lars C. Gormsen, Susanne Keiding, Ole L. Munk, Peter Ott, Michael Sørensen

**Affiliations:** 1 Department of Hepatology and Gastroenterology, Aarhus University Hospital, Aarhus, Denmark; 2 Department of Nuclear Medicine & PET Centre, Aarhus University Hospital, Aarhus, Denmark

**Keywords:** Hepatobiliary kinetics, Positron emission tomography, Liver physiology, ^11^C-CSar, [*N*-methyl-^11^C]cholylsarcosine, BSEP, bile salt export pump, CT, computed tomography, FGF-19, fibroblast growth factor 19, FXR, farnesoid X receptor, ICG, indocyanine green, liver-VOI, liver volume of interest, NTCP, Na^+^ taurocholate co-transporting polypeptide, PET, positron emission tomography

## Abstract

**Background & Aims:**

It is not known how hepatic bile acids transport kinetics changes postprandially in the intact liver. We used positron emission tomography (PET)/computed tomography (CT) with the tracer [*N*-methyl-^11^C]cholylsarcosine (^11^C-CSar), a synthetic sarcosine conjugate of cholic acid, to quantify fasting and postprandial hepatic bile acid transport kinetics in healthy human participants.

**Methods:**

Six healthy human participants underwent dynamic liver ^11^C-CSar PET/CT (60 min) during fasting and from 15 min after ingestion of a standard liquid meal. Hepatobiliary secretion kinetics of ^11^C-CSar was calculated from PET data, blood samples (arterial and hepatic venous) and hepatic blood flow measured using indocyanine green infusion.

**Results:**

In the postprandial state, hepatic blood perfusion increased on average by 30% (*p* <0.01), and the flow-independent hepatic intrinsic clearance of ^11^C-CSar from blood into bile increased by 17% from 1.82 (range, 1.59–2.05) to 2.13 (range, 1.75–2.50) ml blood/min/ml liver tissue (*p* = 0.042). The increased intrinsic clearance of ^11^C-CSar was not caused by changes in the basolateral clearance efficacy of ^11^C-CSar but rather by an upregulated apical transport, as shown by an increase in the rate constant for apical secretion of ^11^C-CSar from hepatocyte to bile from 0.40 (0.25–0.54) min^−1^ to 0.67 (0.36–0.98) min^−1^ (*p* = 0.03). This resulted in a 33% increase in the intrahepatic bile flow (*p* = 0.03).

**Conclusions:**

The rate constant for the transport of bile acids from hepatocytes into biliary canaliculi and the bile flow increased significantly in the postprandial state. This reduced the mean ^11^C-CSar residence time in the hepatocytes.

**Lay summary:**

Bile acids are important for digestion of dietary lipids including vitamins. We examined how the secretion of bile acids by the liver into the intestines changes after a standard liquid meal. The transport of bile acids from liver cells into bile and bile flow was increased after the meal.

## Highlights

• Following a meal, the active transport of bile acids from hepatocytes into bile is increased significantly.
• A meal also increases bile flow out of the liver.
• The postprandial changes are induced shortly after intake of a meal.

## Graphical abstract

**Figure:**
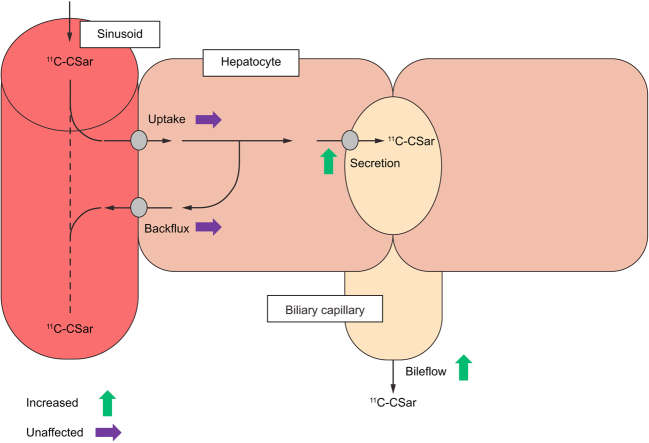

## Introduction

Bile acids are produced in the hepatocytes and secreted across the apical membrane into the biliary canaliculi by active transport^1^ and flow with bile into the gut. Here, bile acids are important for absorption of dietary lipids and lipophile substances.^2^ The intestinal uptake of bile acids, which primarily takes place in the terminal ileum, is highly efficient and facilitated by active transmembrane transport proteins.^3^ After absorption, the bile acids are returned by the portal venous system to the liver where they are removed from the blood and subsequently secreted into the biliary tree. This enterohepatic circulation of bile acids depends strongly on an intact and adaptive transhepatic bile acid transport and secures a high bile acid concentration in the gut with little *de novo* synthesis.^4^^,^^5^

In view of the physiological importance of bile acids, surprisingly little is known about how food intake affects the hepatobiliary bile acid transport kinetics. Soloway and Schoenfield^6^ collected bile from patients through a T-tube catheter placed in the common bile duct after cholecystectomy and reported a 40% increase in hepatic bile acid secretion and a 46% increase in bile flow after a morning meal. Based on intravenously administered radiolabelled bile acids, they further concluded that the increase was caused by increased transhepatic transport of bile acids from blood to bile rather than by hepatic *de novo* synthesis. However, recent surgery to the biliary system with cholecystectomy together with the collection of bile from an invasive catheter that may disturb the enterohepatic circulation of bile acids could have altered the physiology of bile formation and flow.

Positron emission tomography (PET) with the radiolabelled synthetic sarcosine conjugate of cholic acid [*N*-methyl-^11^ C]cholylsarcosine (^11^C-CSar) enables *in vivo* quantification of the separate steps of the hepatobiliary transport of bile acids from blood to bile by external detection and thus without any confounding effects of invasive procedures.[7], [8], [9], [10] In the present study, we used PET to quantify the hepatobiliary secretion kinetics of ^11^C-CSar in healthy human participants before and after intake of a standard liquid meal. The aim of the study was to assess postprandial changes in transhepatic transport of ^11^C-CSar and to examine whether these changes could be related to altered kinetics of the sinusoidal (basolateral) uptake and/or biliary (apical) secretion of the tracer.

## Methods

### Human participants

Six healthy participants (2 women and 4 men, mean age 27 years) with no history of liver disease or daily intake of drugs were included through internet advertisement (Table 1). Routine blood tests including alanine aminotransferase, alkaline phosphatase, bilirubin, gamma-glutamyltransferase, sodium, potassium, albumin, creatinine, C-reactive protein, leucocytes, haemoglobin, thrombocytes, international normalised ratio, glucose, and activated partial thromboplastin time were measured on the day of the experiment and were normal in all participants.

**Table 1:** **Baseline characteristics of the participants**.

Participant ID	Sex	Age (year)	Body weight (kg)	Liver volume (L)	Fasting hepatic blood flow (L blood/min)	Postprandial hepatic blood flow (L blood/min)
1	F	31	82	1.24	1.20	1.70
2	M	26	79	1.25	1.04	1.40
3	M	37	80	1.41	1.15	1.43
4	M	28	95	1.44	1.13	1.58
5	F	18	57	1.12	1.02	1.29
6	M	23	87	1.28	1.51	2.04

F, female; M, male.

### Ethics

The study was approved by the Central Denmark Region Committees on Health Research Ethics (1-10-72-272-16) and conducted in accordance with the Helsinki II Declaration. Written informed consent was obtained from all participants. Pregnancy was ruled out by a negative human chorionic gonadotropin urine test in all female participants. The participants were compensated, receiving 1,000 Danish kroner (approximately 130 euro) for participation. No complications to the procedures were observed. The maximum radiation dose received by the participants was 4.3 mSv.

### Study design

The experiments were conducted in the morning at the PET Centre after an overnight fast (minimum 8 h). First, catheters (Venflon, Becton Dickinson, Kongens Lyngby, Denmark) were placed in both antebrachial veins for administration of the ^11^C-CSar tracer and indocyanine green (ICG), as well as in a hepatic vein via the right femoral vein (Torcon Advantage, Cook Inc., Bjaeverskov, Denmark) and in a radial artery for blood sampling (Artflon, Becton Dickinson, Kongens Lyngby, Denmark).

The first 60-min ^11^C-CSar PET scan was performed with the participant fasting. To avoid signal interference from the first to the second scan, the participant rested for 1 h after the end of the first scan. After this rest, the participant ingested a 500-ml standard liquid meal based on egg and milk produced by the central kitchen at Aarhus University Hospital on the day of the experiment. The energy content was 4,291 kJ, with 1,417 kJ from protein (33%), 1,394 kJ from fat (32%), and 1,468 kJ from carbohydrates (35%). Previous studies have shown that the hepatic blood flow increases shortly after ingestion of a meal before slowly reverting back to normal over the following hours.[11], [12], [13] Therefore, the second 60-min ^11^C-CSar PET scan was initiated 15 min after ingestion of the standard meal.

### ^11^C-CSar PET/CT recording

The PET/computed tomography (CT) studies were performed using a Siemens Biograph 64 Truepoint PET/CT camera (Siemens AG, Ballerup, Denmark). Participants were studied in the supine position, and a tomogram was used to place the individual so that the liver and the bile ducts were in the centre of the 21-cm field of view of the PET camera. A mean dose of ^11^C-CSar 129 MBq (range, 59–205 MBq) was injected intravenously during the initial 20–30 s of a 60-min PET recording. ^11^C-CSar was produced on-site at the Department of Nuclear Medicine & PET Centre as previously described.^14^ PET data were recorded in list mode and reconstructed using attenuation-weighted ordered-subset expectation maximisation with resolution recovery (TrueX) with 4 iterations, 21 subsets, a 336 × 336 × 109 matrix, and a 2-mm Gauss filter. The final PET image voxel size was 2 × 2 × 2 mm^3^, and the time frame structure was 9 × 10 s, 10 × 45 s, and 17 × 3 min. PET measurements were corrected for radioactive decay back to the start of the PET recording.

PET images were analysed using the PMOD software (PMOD Technologies Ltd, Zürich, Switzerland). Regions of interest were drawn in the liver tissue in adjacent planes and combined to produce a volume of interest that was used to generate the time course of the liver tissue concentration of ^11^C-CSar. The total liver volume (*V*_liver_) was estimated using the iso-contour tool from the time period after arterial peak had passed (approximately 70 s) and before ^11^C-CSar appeared in the extrahepatic bile ducts (approximately 180 s). In this period, the hepatic concentration of ^11^C-CSar was greater than the arterial concentration, and the threshold for including the hepatic regions was set to 10% above the arterial peak value in this period.

### Hepatic blood flow

Hepatic blood flow (ml blood/min) was measured during both PET scans using a constant intravenous infusion of ICG, measurements of plasma concentrations of ICG in arterial and hepatic venous blood, and Fick’s principle and corrected for non-steady state.^15^^,^^16^ The hepatic blood perfusion, *Q* (ml blood/min/ml liver tissue), was calculated as the hepatic blood flow divided by *V*_liver_.

### Hepatobiliary ^11^C-CSar kinetics

During both PET recordings, blood samples were taken from the radial artery and hepatic vein (more frequently in the beginning than later during the scan) for measurements of the blood concentration of ^11^C-CSar.^14^ The concentration of ^11^C-CSar in portal venous blood was calculated from the arterial blood concentration using a model for the transfer of tracer through the splanchnic bed.^9^^,^^17^ This model assumes no loss or gain of tracer in the prehepatic splanchnic bed and includes a tracer specific time constant, β, which was estimated for ^11^C-CSar in fasting pigs to a mean value of 13 s (95% CI, 6.0–18.0 s).^8^ The flow-weighted mixed input of ^11^C-CSar to the liver, *C*_in_(*t*), was calculated from the arterial and the modelled portal venous concentrations of ^11^C-CSar using a hepatic arterial flow fraction of 0.25.^18^^,^^19^

**Fig. 1:**
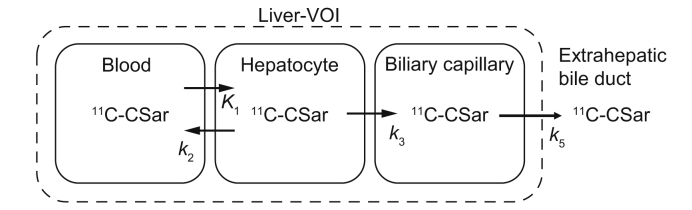
Kinetic compartmental model of the hepatic transport of ^11^C-CSar. The model describes compartments of blood, liver tissue, and intrahepatic bile within the liver-VOI. The exchange of ^11^C-CSar between the compartments is described by rate constants: *K*_1_, unidirectional clearance of ^11^C-CSar from blood to hepatocytes (ml blood/min/ml liver tissue); *k*_2_, rate constant for the backflux from hepatocytes to blood (min^−1^); *k*_3_, rate constant for secretion from hepatocytes to intrahepatic bile (min^−1^); and *k*_5,_ rate constant for flow of ^11^C-CSar to extrahepatic bile (min^−1^). ^11^C-CSar, [*N*-methyl-^11^C]cholylsarcosine; liver-VOI, liver volume of interest.

The kinetic analysis was based on the model illustrated in Fig. 1 and provided quantitative description of the different steps of the hepatic transport of ^11^C-Sar from blood to bile, as previously described.^9^^,^^20^

The hepatic extraction fraction, *E*(*t*), was calculated using C_in_(*t*) and the output concentration of ^11^C-Csar in the hepatic vein, *C*_out_(*t*). *C*_out_(*t*) was corrected for non-steady state using individual estimates of mean hepatic blood transit time, *T*, calculated as the difference between the peak time of the ^11^C-CSar concentration in hepatic input and output.

(1) $$E\left(t\right)=\frac{C_{\text{in}}\left(t\right)-C_{\text{out}}\left(t+T\right)}{C_{\text{in}}\left(t\right)}$$

The initial extraction fraction, *E*_0_, was calculated using the recordings of the first minute (first pass) after the bolus injection where potential backflux of ^11^C-CSar from hepatocytes to blood can be ignored.

The AUCs of *C*_in_(*t*) and *C*_out_(*t*) for the first 50 min after administration of ^11^C-CSar were used to calculate *E*_AUC_, which was used as a measure of the steady-state hepatic extraction fraction.^9^

The permeability surface area product of the hepatocyte plasma membrane, *PS*_mem_, which is a measure of the efficacy of the transport of bile acids across the sinusoidal membrane from blood to hepatocytes, was calculated as^21^

(2) $$PS_{\text{mem}}=-Q\;\ln\left(1-E_{0}\right)$$

The flow-independent intrinsic clearance, *Cl*_int_, which is a measure of the overall hepatic transport efficacy from blood to bile, was calculated as^22^

(3) $$Cl_{\mathrm{int}}=-Q\text{ ln }\left(1-E_{\text{AUC}}\right)$$

The kinetic model shown in Fig. 1 consists of well-mixed compartments of blood, hepatocytes, and intrahepatic bile ducts. The unidirectional clearance of ^11^CSar from blood into hepatocyte, *K*_1_, and the rate constants *k*_2_, *k*_3_, and *k*_5_ for the transport between the compartments were estimated by nonlinear regression using *C*_in_(*t*) as input function and hepatic tissue concentration as output function. Using the previously published normal values for the rate constants as a starting point in the regression, the fitting was consistent and reproducible without the use of further assumptions.^9^^,^^20^ This enabled estimation of rate constants for the separate transport steps and calculation of mean hepatocyte residence time of ^11^C-Csar^9^:

(4) $$T_{\text{hep}}=1/\left(k_{2}+k_{3}\right)$$

*T*_hep_ is the average time ^11^C-CSar molecules reside in the hepatocytes before being either secreted into bile (*k*_3_) or transported back to blood (*k*_2_). For *k*_3_ ≫ *k*_2_, *T*_hep_ characterises the time a ^11^C-CSar molecule uses to traverse the hepatocyte from blood to bile. We also calculated bile flow (*F*_bile_ = *k*_5_*V*_liver_*V*_bile_, where *V*_liver_ is the total liver volume as defined above and *V*_bile_ is the fraction of intrahepatic bile ducts in the liver tissue) and concentration ratios between blood, hepatocyte, and bile, as previously described.^9^

### Endogenous bile acids and fibroblast growth factor 19

Before and after each of the 2 PET scans, plasma concentrations of endogenous bile acids and fibroblast growth factor 19 (FGF-19) were measured in blood samples from the radial artery and hepatic vein. During the postprandial examination, endogenous bile acids and FGF-19 were also measured twice during the PET scan (at 20 and 40 min). Endogenous bile acids were measured in plasma at the Department of Clinical Biochemistry at Aarhus University Hospital, Denmark. FGF-19 was measured in plasma using ELISA according to the manual of the manufacturer (Human FGF-19 Quantikine ELISA Kit, R&D Systems, Minneapolis, Minnesota, USA).

### Statistical analysis

All data were normally distributed and are therefore presented as mean (95% CI). Comparisons between baseline and postprandial state were examined using paired *t* tests, and a *p* value <0.05 was considered to indicate a statistically significant difference between the fasting and postprandial states. Statistical analyses were performed using STATA software, release 13 (StataCorp, College Station, TX, USA). The number of participants was based on previous experience from functional PET studies.

## Results

**Fig. 2:**
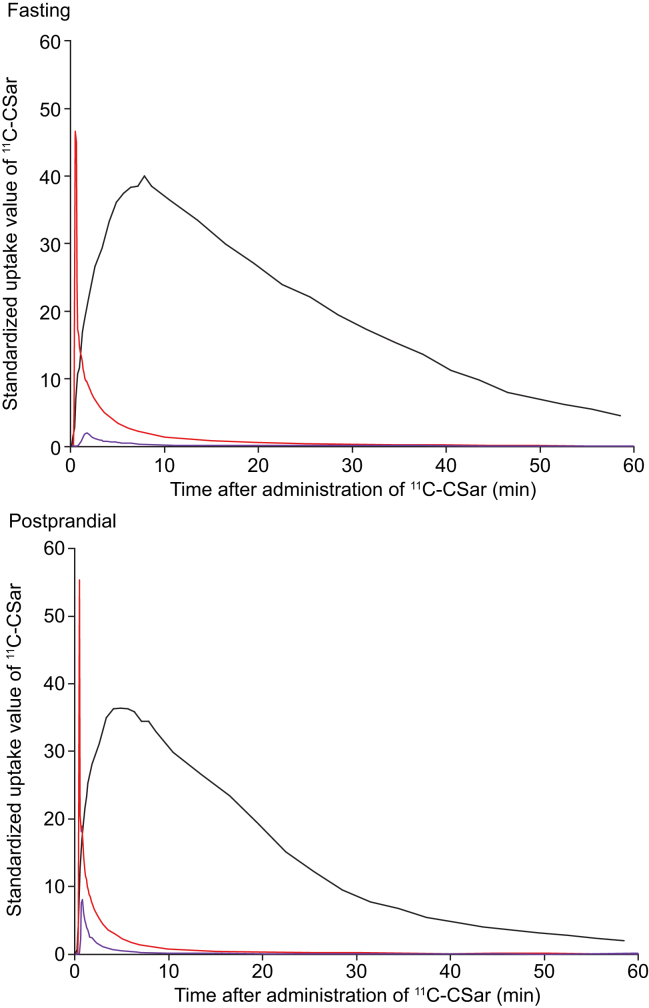
Time courses of the ^11^C-CSar concentration in arterial blood (red), hepatic venous blood (purple), and liver tissue (black) in the fasting and postprandial states. ^11^C-CSar concentration are shown as standardized uptake values, where the concentrations are corrected for the individual dose of ^11^C-CSar and body weight. ^11^C-CSar, [*N*-methyl-^11^C]cholylsarcosine.

Fig. 2 shows examples of the time courses of the concentrations of ^11^C-CSar in the fasting and postprandial states in the same participant standardised for injected dose of ^11^C-CSar and body weight. The concentration of ^11^C-CSar in the liver tissue increased rapidly after ^11^C-CSar administration and peaked at comparable levels in the fasting and postprandial states, whereas the subsequent decrease, representing hepatobiliary secretion and bile flow out of the liver, was more rapid after food intake than during fasting. In the postprandial state, hepatic venous blood had a higher concentration of ^11^C-CSar, likely as a result of increased hepatic blood flow, but not enough to affect the hepatic extraction fractions as described in the next section.

### Kinetics for the uptake of ^11^C-CSar from blood to hepatocyte

Neither *E*_0_ nor *E*_AUC_ was statistically significantly different when comparing the postprandial and fasting states (Table 2), although there was a trend towards a decrease in *E*_AUC_ (*p* = 0.054). Mean *PS*_mem_ was 3.72 (2.21–5.22) ml blood/min/ml liver tissue in the fasting state and not statistically significantly different from 4.08 (2.36–5.80) ml blood/min/ml liver tissue in the postprandial state (*p* = 0.51). *PS*_mem_ was more than 3 times higher than hepatic blood perfusion, typical for high extraction compounds and underlining the efficient transport of ^11^C-CSar from blood to hepatocyte.

**Table 2:** **Hepatobiliary transport kinetics**.

Parameter	Fasting value (mean [95% CI])	Postprandial value (mean [95% CI])	*p* value postprandial *vs*. fasting (paired *t* test)
*Q* (ml blood/min/ml liver tissue)	0.92 (0.76–1.07)	1.23 (1.00–1.45)	**<0.01**
*E*_0_	0.97 (0.92–1.00)	0.94 (0.87–1.00)	0.39
*E*_AUC_	0.86 (0.81–0.91)	0.82 (0.74–0.90)	0.054
*PS*_mem_ (ml blood/min/ml liver tissue)	3.72 (2.21–5.22)	4.08 (2.36–5.80)	0.51
*Cl*_int_ (ml blood/min/ml liver tissue)	1.82 (1.59–2.05)	2.13 (1.75–2.50)	**0.042**
*K*_1_ (ml blood/min/ml liver tissue)	0.95 (0.66–1.23)	1.24 (0.87–1.61)	**<0.01**
*k*_2_ (min^−1^)	0.01 (0.0–0.02)	0.003 (0.0–0.01)	0.55
*k*_3_ (min^−1^)	0.40 (0.25–0.54)	0.67 (0.36–0.98)	**0.034**
*k*_5_ (min^−1^)	0.07 (0.05–0.10)	0.10 (0.08–0.12)	**0.048**
*T*_res_ (min)	2.79 (1.57–4.00)	1.90 (0.57–3.24)	**0.033**
Bile flow (ml bile/min)	0.30 (0.21–0.40)	0.40 (0.34–0.46)	**0.044**
*C*_hep_/*C*_in_	3.66 (2.65–4.67)	3.31 (1.47–5.14)	0.45
*C*_bile_/*C*_hep_	1,163 (693–1,632)	1,453 (724–2,181)	0.41

Values in bold denote statistical significance. Q, hepatic blood perfusion (ml blood/min/ml liver tissue); *E*_0_, unidirectional hepatic extraction fraction of ^11^C-CSar; *E*_AUC_, hepatic extraction fraction of ^11^C-CSar using AUC of input and output at 50 min; *PS*_mem_, permeability surface area product of ^11^C-CSar (ml blood/min/ml liver tissue); *Cl*_int_, hepatic intrinsic clearance of ^11^C-CSar (ml blood/min/ml liver tissue); *K*_1_, unidirectional clearance of ^11^C-CSar from blood to hepatocytes (ml blood/min/ml liver tissue); *k*_2_, rate constant for backflux of ^11^C-CSar from hepatocytes to blood (min^−1^); *k*_3_, rate constant for the secretion of ^11^C-CSar from hepatocytes to bile (min^−1^); *k*_5_, rate constant for flow of ^11^C-CSar in bile out of the liver-VOI (min^−1^); *T*_hep_ (min), which is the average time ^11^C-CSar molecules reside in the hepatocytes; *C*_hep_/*C*_in_, the ratio between the steady-state concentration of ^11^C-CSar in hepatocytes and blood; *C*_bile_/*C*_hep_, the ratio between intrahepatic bile and hepatocytes; ^11^C-CSar, [*N*-methyl-^11^C]cholylsarcosine; liver-VOI, liver volume of interest.

As shown in Table 2, mean hepatic blood perfusion increased by 30% in the postprandial state (*p* <0.01). With a high *PS*_mem_, hepatic clearance of ^11^C-CSar from blood will increase with flow,^22^ and in accordance, *K*_1_, the flow-dependent clearance of tracer from blood to hepatocyte, increased proportionally from 0.95 (0.66–1.23) ml blood/min/ml liver tissue to 1.24 (0.87–1.61) ml blood/min/ml liver tissue in the postprandial state (*p* = 0.003).

The meal did not change *PS*_mem_, but it did increase the flow-independent hepatic intrinsic clearance of ^11^C-CSar from blood to bile (*Cl*_int_) by on average by 17% from a mean baseline value of 1.82 (1.59–2.05) ml blood/min/ml liver tissue to a mean postprandial value of 2.13 (1.75–2.50) ml blood/min/ml liver tissue (*p* = 0.042). This illustrates that the overall transport capacity of ^11^C-CSar from blood to bile increased postprandially, an increase that was independent of the increased hepatic blood flow and explained by an increased apical transport of ^11^C-CSar from hepatocytes to bile canaliculi (see below).

### Kinetics for the transport of bile acids from hepatocyte to bile

**Fig. 3:**
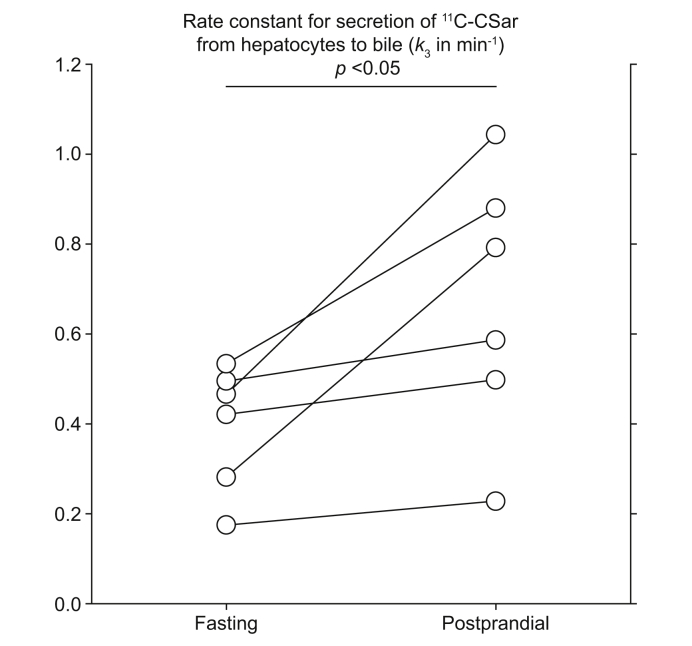
Individual pairs of fasting and postprandial values of *k*_3_, the rate constant for secretion of ^11^C-CSar from hepatocyte to intrahepatic bile (min^−1^). *p* <0.05 (paired *t* test). ^11^C-CSar, [*N*-methyl-^11^C]cholylsarcosine.

As seen in Table 2, the rate constant for backflux of ^11^C-CSar from hepatocyte to blood, *k*_2_, did not change postprandially (*p* = 0.55), whereas the rate constant for apical secretion from hepatocytes to bile, *k*_3_, increased 68% from mean 0.40 (0.25–0.54) min^−1^ to mean 0.67 (0.36–0.98) min^−1^ (*p* = 0.03) (Fig. 3). This is in accordance with the observed increase in *Cl*_int_.

**Fig. 4:**
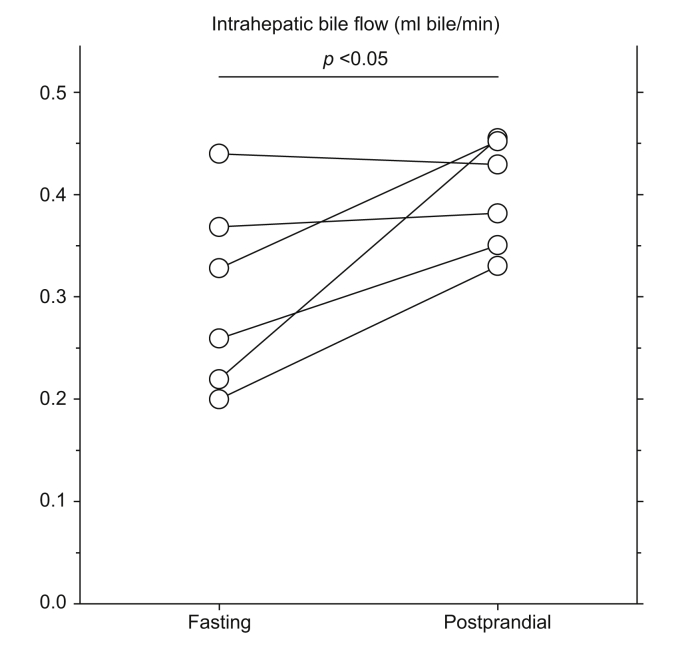
Individual pairs of fasting and postprandial values of intrahepatic bile flow (ml bile/min). *p* <0.05 (paired *t* test).

The intrahepatic bile flow was 0.30 (0.21–0.40) ml bile/min in the fasting state and increased significantly to an average of 0.40 (0.34–0.46) ml bile/min during the postprandial examination (*p* = 0.03) (Fig. 4).

The mean hepatic residence time of ^11^C-CSar in the hepatocytes was 2.79 (1.57–4.00) min during fasting and decreased significantly to 1.90 (0.57–3.24) min in the postprandial state (*p* = 0.03) because of increased *k*_3_. As shown in Table 2, this secured unchanged concentration ratios between blood and hepatocyte (*p* = 0.45) and between hepatocyte and intrahepatic bile (*p* = 0.41).

### Endogenous bile acids and FGF-19

**Fig. 5:**
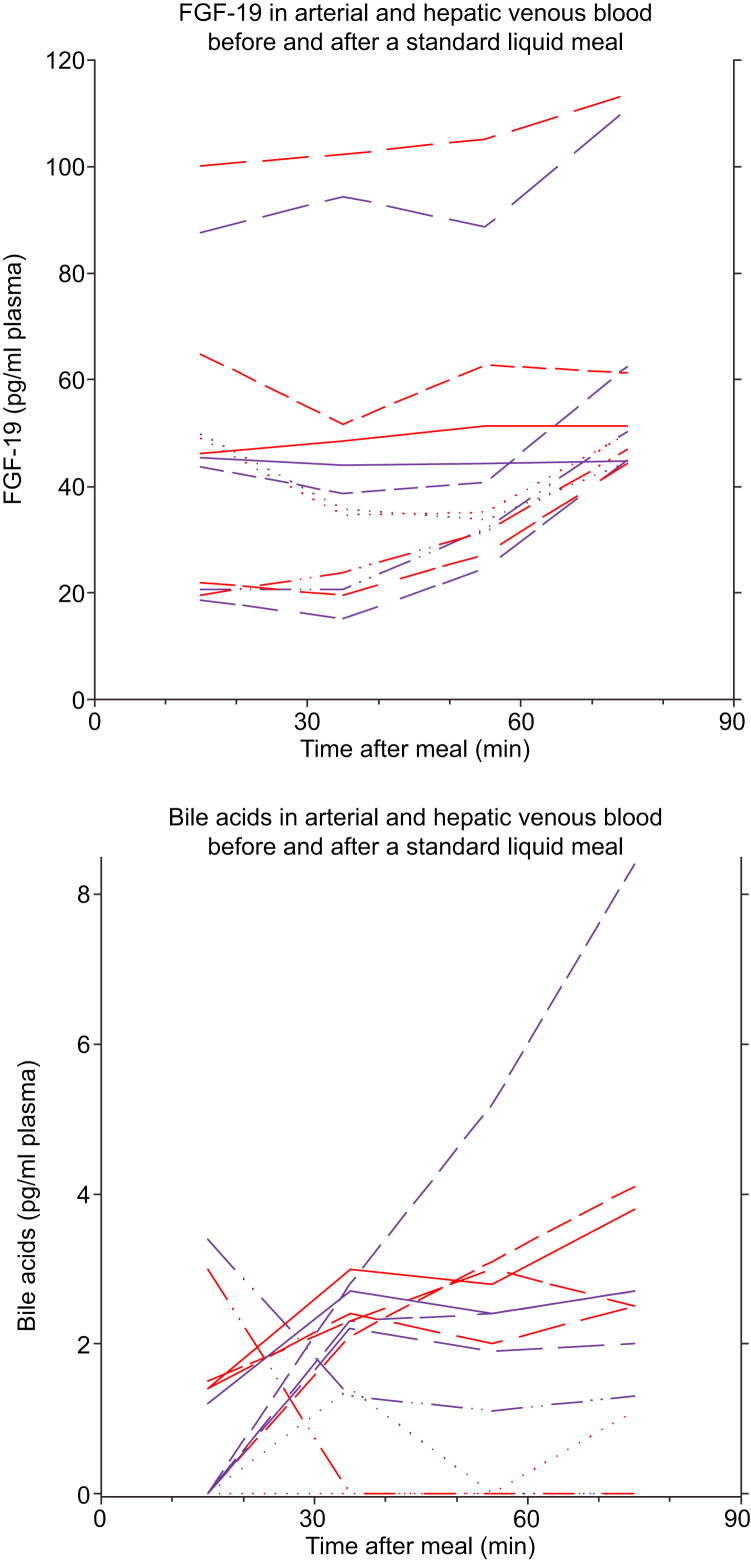
Individual time courses of arterial (red) and hepatic venous (purple) plasma concentrations of FGF-19 (pg/ml plasma) and endogenous bile acids (pg/ml plasma). The meal was ingested 15 min before the start of the postprandial examination. FGF-19, fibroblast growth factor 19.

Fig. 5 shows a large interindividual variation in the postprandial plasma concentrations of bile acids and FGF-19. As no differences were found between the 2 concentrations measurements of FGF-19 during fasting (at the start and the end of the fasting experiment, *p* >0.10 for all), the mean of the 2 fasting values (venous and arterial separately) were used as a baseline for comparison with postprandial values. In the postprandial state, the concentration of endogenous bile acids increased significant after 20 min, whereas only the 60-min value was significantly different for FGF-19 (Fig. 5).

## Discussion

This is the first *in vivo* quantification of the postprandial changes in the hepatobiliary transport of bile acids from blood to bile. The main observation was an increase in the apical transport rate constant (*k*_3_) by two-thirds in the postprandial state as compared with the fasting state. This occurred during the period 15 min to 1.25 h after food intake and significantly reduced the mean hepatic residence time of bile acids.

The hepatobiliary kinetics during fasting (Table 2) were in the same order of magnitude as our previously published data, and with low interindividual variability, indicating the stability of the methodology used and described in detail elsewhere.^9^^,^^20^

Fasting *PS*_mem_ was high and did not change after the meal, and in accordance with this, *E*_*o*_ was close to 1.0 both before and after the meal. Interestingly, *Cl*_int_ increased in the postprandial state (from 1.82 to 2.13 ml blood/min/ml liver tissue) which suggests an increase in the transport capacity of bile acids from hepatocytes into biliary capillaries briefly after a meal as quantified by the rate constant for secretion, *k*_3_, which increased from a mean fasting value of 0.40 min^−^^1^ to a mean postprandial value of 0.67 min^−^^1^. This means that in the postprandial state, 67% of hepatocellular ^11^C-CSar was secreted into biliary capillaries every minute in contrast to only 40% during fasting. The increase in *k*_3_ reduced the hepatic residence time so that the average ^11^C-CSar molecule was 1.9 min (0.57–3.24 min) to traverse the hepatocyte in the postprandial state, in contrast to 2.79 min (1.57–4.00 min) in the fasting state. In accordance with bile flow being largely regulated by bile acids,^23^ the rate constant for bile flow, *k*_5_, increased from 0.07 to 0.10 min^−^^1^ and the calculated bile flow from 0.30 to 0.40 ml bile/min. Taken together, these observations illustrate a rapid postprandial recruitment of an apical transport reserve in the healthy liver preventing hepatic bile acid accumulation and securing delivery of bile acids to the intestines.

The present study does not allow for mechanistic conclusions on how the changes relate to potential changes in specific transporter proteins. However, the basolateral transport of ^11^C-CSar, mainly facilitated by Na^+^ taurocholate co-transporting polypeptide (NTCP/*SLC10A1*),^24^ did not change in the postprandial state, which is in accordance with a very high capacity of this transport protein, allowing for efficient extraction of bile acids from the sinusoidal blood even under conditions with very high concentration of bile acids returning to the liver. The rapid increase in the apical bile acid transport from hepatocyte to bile, facilitated by the bile salt export pump (BSEP/*ABCB11*),^25^ is most likely mediated by an increased expression of BSEP proteins on the apical membrane. Experimental studies have shown that different substrates such as ursodeoxycholic acid, tauroursodeoxycholic acid, and 4-phenylbutyrate increase the expression of BSEP on the apical membrane, most likely by recruitment from subcanalicular intracellular vesicles.[26], [27], [28], [29], [30] This effect is thought to be mediated by a nuclear receptor, farnesoid X receptor (FXR), which can be stimulated directly by bile acids.[31], [32], [33] FGF-19, which is released to portal blood when bile acids stimulate FXR in the ileocytes, increased in both arterial and hepatic venous blood at the end of the postprandial scan but is likely to have increased in portal blood earlier.

The postprandial intestinal uptake of bile acids may also increase the concentration of bile acids in the portal blood, but because the transit time for bile acids through the small intestines is more than 2 h in healthy humans, there is a rather large window before a significant circulation of ^11^C-CSar is expected to be observable.^34^^,^^35^ Moreover, recirculation of the tracer would primarily be observable in portal venous blood, which we could not sample in the present study. In an invasive pig study performed during fasting, a significant increase in portal venous concentration of ^11^C-CSar did not occur until after 75 min.^7^

Soloway and Schoenfield,^6^ using an invasive setup with bile sampling from a T-tube in the common bile duct following cholecystectomy, observed a postprandial increase in bile flow of 40% and bile salt secretion by 46%. Though their experimental setup interfered with the enterohepatic circulation of bile acids, Soloway and Schoenfield^6^ also suggested that the increased biliary secretion of bile acids was caused by increased enterohepatic recirculation rather than by increased *de novo* synthesis. Our study, which does not interfere with the enterohepatic circulation, validates their findings and interpretations by a direct quantification of the separate transport steps involved in hepatic uptake and secretion of bile acids as well as bile flow.

In conclusion, the transhepatic transport of bile acids increased in the postprandial state compared with that in fasting, and our data show that this was mainly because of an increased apical bile acid secretion. The mechanism is not clear, but we hypothesise that it is driven by increased apical expression of BSEP. Further studies may clarify whether the rapid change could be explained by an increased apical transporter expression as a result of recruitment of internalised vesicular transporters or if other mechanisms are involved.

## Financial support

This work was supported by the 10.13039/501100010983Health Research Fund of Central Denmark Region, Denmark, 10.13039/501100003035Aase and Ejnar Danielsen’s Foundation, Denmark (10-001895), the 10.13039/100008392Danish Council for Independent Research (Medical Sciences, 8020-00427B), and Aage and Johanne Louis-Hansen’s Foundation (17-2-0196).

## Authors’ contributions

Study concept: NWØ, SK, PO, MS. Obtained funding: NWØ. Acquisition of data: NWØ, LCG, SK. Analysis of data: NWØ, SK, OLM, PO, MS. Statistical analysis: NWØ. Drafting of manuscript: NWØ, PO, MS.

Critical revision of manuscript: NWØ, LCG, SK, OLM, PO, MS. Approved the final manuscript: All authors.

## Data availability statement

The datasets generated during and/or analysed during the current study are available from the corresponding author on reasonable request.

## Conflicts of interest

The authors have nothing to disclose.

Please refer to the accompanying ICMJE disclosure forms for further details.
